# A collaborative application for characterizing colorectal lesions could improve quality of tumor resection

**DOI:** 10.1055/a-2208-2863

**Published:** 2023-12-11

**Authors:** Pierre Lafeuille, Orlando Chuquimia, Clara Yzet, Jérémie Jacques, Victoria Nurcelli, Jérôme Rivory, Mathieu Pioche

**Affiliations:** 1 Department of Endoscopy and Hepatogastroenterology, Pavillon L, Edouard Herriot Hospital, Lyon, France; 2 Echopen Factory, Paris, France; 3 27063 Department of Systems on Chip, Sorbonne University, Paris, France; 4 Department of Gastroenterology, Amiens University Hospital, Amiens, France; 5 Department of Gastroenterology and Endoscopy, Dupuytren University Hospital, Limoges, France

Accurate endoscopic characterization of colorectal lesions is essential for predicting histology and choosing the most appropriate resection technique, but it remains very difficult for endoscopists 1 . Lesions are characterized in real time according to their macroscopic appearance, vascular pattern, and pit pattern, in white light and virtual chromoendoscopy. Numerous classifications are required to fully characterize the various colorectal lesions, but few gastroenterologists are familiar with the classifications or use them in daily practice 1 .

Therefore, we have integrated all the validated criteria into a single table, the CONECCT classification ( Fig. 1 ), enabling prediction of both histology and appropriate treatment strategy 1 2 3 . To further improve the level of characterization of French gastroenterologists, particularly residents, it would be necessary to give as many of them as possible access to a community of gastroenterologists, including experts, for external characterization advice.

**Fig. 1:**
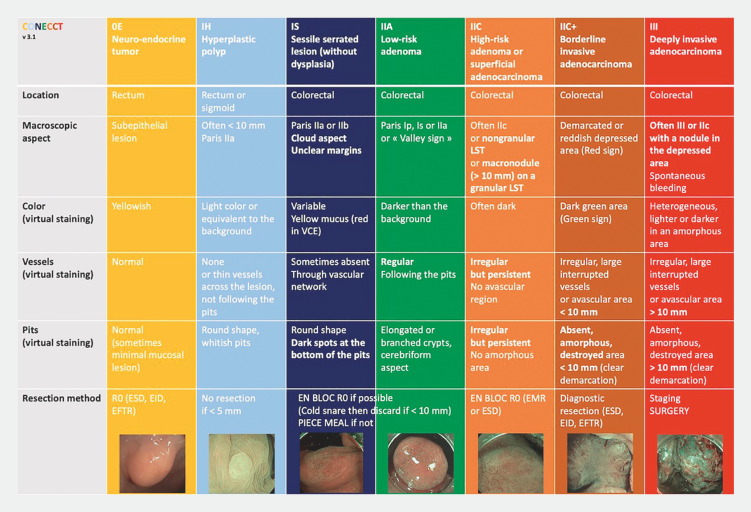
The CONECCT Classification (version 3.1). EFTR, endoscopic full-thickness resection; EID, endoscopic intermuscular dissection; EMR, endoscopic mucosal resection; ESD, endoscopic submucosal dissection; LST, laterally spreading tumor; VCE, virtual chromoendoscopy.

We report here the case of a colorectal lesion characterized using the “CONECCTapp” application, a free collaborative Smartphone application, supported by the Société Française d’Endoscopie Digestive (SFED).

Faced with a difficult colorectal lesion, a resident was able to submit photos of the lesion and complete its characteristics while being guided by the application at each stage. This request was then visible to all gastroenterologists with access to the application, including experts, who could characterize the lesion in return, based on the information provided ( Fig. 2 , Video 1 ).

**Fig. 2:**
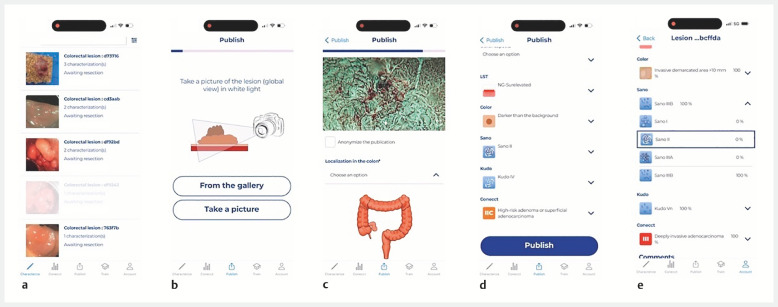
Use of the CONECCTapp. **a** View of the current list of lesions submitted to the community on the “CONECCTapp” application. **b** The application guides the user when publishing a lesion. **c** Photo of the lesion by the user. **d** Lesion characterization by the user. **e** Display of the expert’s characterization results in percentages (user initial choice in the blue rectangle).

Example of the use of a collaborative application for characterization of a difficult colorectal lesion.Video 1

This application could help gastroenterologists to establish a faster and more accurate diagnosis during colonoscopy and improve the quality of tumor resection. In a second phase, and to further reduce response time, we plan to integrate an artificial intelligence module for automatic characterization. Furthermore, this device could improve patient care wherever they are treated and reduce stress for doctors by giving them access to expert advice. Further clinical studies on the positive impact of such a tool are planned.

Endoscopy_UCTN_Code_TTT_1AU_2AB

## Notes

Endoscopy E-Videos https://eref.thieme.de/e-videos
*E-Videos* is an open access online section of the journal *Endoscopy* , reporting on interesting cases and new techniques in gastroenterological endoscopy. All papers include a high-quality video and are published with a Creative Commons CC-BY license. Endoscopy E-Videos qualify for HINARI discounts and waivers and eligibility is automatically checked during the submission process. We grant 100% waivers to articles whose corresponding authors are based in Group A countries and 50% waivers to those who are based in Group B countries as classified by Research4Life (see: https://www.research4life.org/access/eligibility/ ). This section has its own submission website at https://mc.manuscriptcentral.com/e-videos .
